# Assessment of Oral Chemotherapy Nonadherence in Chronic Myeloid Leukemia Patients Using Brief Measures in Community Cancer Clinics: A Pilot Study

**DOI:** 10.3390/ijerph182111045

**Published:** 2021-10-21

**Authors:** Terry C. Davis, Connie L. Arnold, Glenn Mills, Glenn J. Lesser, W. Mark Brown, Richard Schulz, Kathryn E. Weaver, Pamala A. Pawloski

**Affiliations:** 1 Department of Medicine, Louisiana State University Health—Shreveport, Shreveport, LA 71130, USA; connie.arnold@lsuhs.edu (C.L.A.); glenn.mills@lsuhs.edu (G.M.); 2 Department of Internal Medicine, Wake Forest School of Medicine, Winston-Salem, NC 27101, USA; glesser@wakehealth.edu; 3 Department of Biostatistics and Data Science, Wake Forest School of Medicine, Winston-Salem, NC 27101, USA; wmbrown@wakehealth.edu; 4 College of Pharmacy, University of South Carolina, Columbia, SC 29208, USA; schulz@cop.sc.edu; 5 Department of Social Sciences and Health Policy, Wake Forest School of Medicine, Winston-Salem, NC 27101, USA; keweaver@wakehealth.edu; 6 Metro-Minnesota Community Oncology Research Consortium, St. Louis Park, MN 55416, USA; pamala.a.pawloski@healthpartners.com

**Keywords:** chronic myelogenous leukemia, antineoplastic agents, medication adherence, self-report, health literacy

## Abstract

The purpose of this pilot study was to assess Chronic Myeloid Leukemia (CML) patients’ adherence to, beliefs about, and barriers to oral anticancer agents (OAC) using brief self-report measures in community-based cancer clinics. Patients completed a structured interview including a health literacy assessment, a Brief Medication Questionnaire, two single-item self-report adherence questions, and the Medications Adherence Reasons Scale. Of the 86 participants, 88.4% were white; 55.8% male; mean age, 58.7 years; and 22.1% had limited health literacy. Nonadherence (missing at least one dose in the last week) was reported by 18.6% of participants and associated (*p* < 0.003) with less-than-excellent perceived ability to take CML medications (16.3%). Black participants reported more difficulty taking CML medications than white participants (28.6% vs. 8.3%, *p* = 0.053). Among all participants, 43.0% reported their CML medicine was ineffective and 24.4% that taking CML pills was somewhat to very hard. The most common reasons for missing a dose were simply missed it (24.4%) and side effects (18.6%). Most patients perceived their ability to take CML medication was good to excellent, yet nearly one in five reported missing at least one dose in the last week. Brief, no-cost self-report assessments to screen CML patients’ OAC adherence, barriers, and beliefs could facilitate counseling in busy community cancer clinics.

## 1. Introduction

Oral anticancer agent (OAC agent) use is increasing and accounts for an estimated one-third of cancer drugs under development [1,2]. Oral antineoplastic therapies can improve survival, lower treatment-associated costs, and decrease patient burden by minimizing clinic visits or eliminating the need for infusions [3]. Despite the advantages, references [1,4,5,6,7,8,9,10] nonadherence to OAC agents has become a significant problem in modern oncology treatment.

The World Health Organization (WHO) reported that nonadherence to oral medication is the single most modifiable factor in treatment outcomes and has a greater impact than improvement in treatments [10]. Optimal medication adherence has been defined as a patient taking their medication exactly as prescribed, at the exact time, the correct dosage, and for the recommended length of time [11,12]. However, one standard definition for medication adherence by which all measures are compared is lacking [13]. 

Oral tyrosine kinase inhibitors (TKIs) have transformed chronic myeloid leukemia (CML) from a fatal disease to a chronic illness [14]. The National Cancer Institute estimates there will be 9110 new cases of CML in 2021 with a five-year survival rate of 70.6% [15]. 

Survival benefits can only be realized if patients with CML consistently adhere to their medications [16,17,18]. A minimum threshold of 85% adherence has been described as critical to maintaining a complete cytogenic response in CML [18]. Yet, several recent studies have found a 25–35% prevalence of nonadherence to oral CML therapy [19]. Many of these studies defined adherence as use of 85–90% of the prescribed drug [20]. However, a recent systematic review identified nineteen studies reporting prevalence rates of oral chemotherapy adherence in adult CML patients ranging from 76% to 98% [21]. Reports of patients who are fully (100%) adherent only range from 20% to 53% [22,23,24]. The wide variation is due to differences in adherence definitions, measures, and the type of education patients received [21].

Most CML adherence studies were performed in a single center using pharmacy claims data [25], investigator pill counts [26], patient treatment diary templates [27], electronic monitoring devices [17,28], or physician surveys [29]. Few studies have also included self-report assessments. Krikorian et al., used the Beliefs about Medicine Questionnaire, which had been used in chronic disease studies to assess patients with cancer or a higher risk for cancer [26]. It overestimated oral chemotherapy adherence compared to nurse or pharmacist pill counts. Daouphars et al. developed a 10-item adherence self-report and compared it to prescription refills to measure imatinib adherence [25].

Some self-report studies used web-based surveys. Buzaglo et al. used a 51-item self-report [19] that assessed patient characteristics, financial burden, psychosocial distress, and the medication adherence of patients with CML participating in the Cancer Experience Registry. Geissler et al. [30] used the eight-item Morisky Medication Adherence Scale [31] to assess TKI adherence in patients participating in the CML Advocates Network. These studies did not assess adherence in underserved populations or health literacy, which is more prevalent in low-income and minority populations. Better understanding of OAC agent adherence among the underserved and those with low health literacy is needed [32]. 

Single item assessments (of missed pills over 7 days or 4 weeks) have been used to successfully assess nonadherence relevant to chronic disease outcomes but have not been used to assess TKI nonadherence [33,34,35]. One single item self-report question assessing missed doses over the past 7 days has been used in general medicine and cardiology outpatient clinics serving patients with limited income and literacy [33]. This multisite study found this single item question was particularly well suited for use in busy clinic settings and served as simple means of identifying at-risk patients for interventions to support adherence.

The purpose of this pilot study (WF 99716CD) was to (1) assess the rate of OAC agent nonadherence among patients diagnosed with CML and treated in community cancer clinics using multiple self-report measures and (2) characterize patients’ barriers and beliefs regarding CML OAC medication adherence. This study was conducted to obtain baseline information to inform future research. We collaborated with multiple community-based oncology practices participating in the National Cancer Institute (NCI) Community Oncology Research Program (NCORP) to enhance the generalizability of study findings to future community oncology clinical settings. 

## 2. Materials and Methods

### 2.1. Setting

Cancer care delivery researchers in the Gulf South Minority/Underserved site of the National Community Oncology Research Program (NCORP) led this study. NCORP is a National Cancer Institute-funded national network to provide cancer clinical trials and care delivery studies in the community setting. The study was coordinated by the Wake Forest NCORP Research Base and conducted at five NCORP network community practices that saw at least 30 patients with CML in the past year and were interested in assessing adherence to OAC agents. Participating practices were well distributed geographically (Northeast, Great Plains, and Southeast) and of varied practice ownership types (e.g., academic medical centers and regional health systems with and without integrated health plans); two of the five practices were connected with Minority/Underserved NCORP Community Sites. The study was conducted from January through April 2017. 

### 2.2. Research Training and Participant Recruitment

NCORP practice study staff completed site-specific CITI training and were trained by the study team on administration of all instruments, data collection from EHRs, and data entry. The staff also piloted the protocol with other research staff before enrolling participants to ensure feasibility of implementation.

NCORP practice study staff reviewed cancer center appointment schedules to identify eligible participants (English-speaking adults with a diagnosis of CML who had been prescribed OAC agents (imatinib, nilotinib, dasatinib, bosutinib, and/or ponatinib) for at least 30 days). Eligible patients were recruited during routine clinic visits during the study period. Structured interviews were conducted to assess basic demographic information, four no-cost brief self-report measures assessed adherence, beliefs about the medication, and potential reasons for nonadherence. A brief health literacy assessment was also administered. The Wake Forest Health Science Institutional Review Board approved the study, including informed consent.

### 2.3. Study Measures

The study measures included medical record access, registration, and structured interviews. To assess potential correlates of OAC agent adherence, medical records were accessed to ascertain data related to patient characteristics (e.g., age, gender, health insurance coverage, co-morbid diseases, and number of non-CML prescriptions). Additional patient characteristics including ethnicity and race were asked in the brief interview developed by the authors. The session was conducted during a routine clinic visit and lasted approximately 15 minutes. We performed in-person interviews to overcome potential barriers associated with health literacy.

### 2.4. Adherence

The Brief Medication Questionnaire (BMQ) is a self-report tool for screening adherence and barriers to adherence of all medications taken during the previous week [36]. We modified the regimen screen to include only the question of how many times did you miss taking a dose of your CML medication in the previous week. We also kept the 2-item Belief Screen that asks, how well does the medication work for you (very well, ok, not well) and does the medicine bother you and a 1-item Recall Screen about potential difficulties remembering to take pills (very, somewhat, not at all). We characterized nonadherence as any score above 0. 

Single-Item Adherence Measure 1. This question asked, “Over the past seven days, how many times did you miss a dose of your CML medication?” This measure (and similarly worded measures) has been used in previous chronic disease studies [33]. We characterized nonadherence as any score above 0. 

Single-Item Adherence Measure 2. The Self-Rating Scale Item is a single question that uses a five-point scale to measure adherence to medication over the last 4 weeks [37,38]. It has demonstrated low patient burden and the ability to predict adherence-related clinical outcomes in HIV patients as good as or better than other adherence measures [37]. This item asks: “Thinking about the past four weeks, please rate your ability to take your CML medicine as prescribed” and it is scored on a five-point Likert scale (Excellent, Very Good, Good, Fair, and Poor). This question has been shown to significantly correlate with other measures of self-reported adherence and a medication event monitoring system (MEMS) [39] in the HIV literature. We considered those self-reporting excellent adherence as adherent and those self-reporting less than excellent as nonadherent. 

The Medications Adherence Reasons Scale (MARS) is an 11-item validated survey that identifies potential risk factors associated with medication adherence [34,35]. Items are based on the frequently reported reasons for nonadherence and are scored on a 5-point Likert scale. It was designed to assess medication adherence in patients with multiple chronic conditions. 

Health Literacy has previously been identified as a barrier to adequate prescription medication adherence but has not been studied with OAC therapy. Health literacy was assessed using the Rapid Estimate of Adult Literacy in Medicine short form (REALM-SF), a validated commonly used 7-item health word reading recognition test [40]. Raw scores range from 0 to 7 and can be converted to reading grade levels: a score of 7 indicates 9th grade or higher which can be interpreted as adequate health literacy. A score of 6 or below is reading below a high school level and can be considered limited health literacy. 

### 2.5. Statistical Analysis

Results are presented as mean (standard deviation), median, or number (percentage) where appropriate. Differences in demographic and clinic variables between adherent and nonadherent participants were assessed using the Kruskal-Wallis test for continuous variables and the chi-square or Fischer’s exact test (where cell counts were low) for categorical variables. A *p*-value of less than 0.05 was considered statistically significant. All analyses were performed using SAS version 9.3 (SAS Institute, Cary, NC, USA) [41]. 

## 3. Results

A total of 86 participants were enrolled. Enrollment ranged from 2 to 30 participants per site. The mean age was 58.7 years, 88.4% were non-Hispanic white, 55.8% were male, 22.1% had limited health literacy (read below a 9th-grade level), and 75.6% were privately insured (Table 1). More than 69.8% had at least one comorbid disease and all participants took at least one non-CML prescription drug; 29.1% took six or more non-CML medications. There was no difference in adherence by demographics; however, participants who reported nonadherence had higher mean BMIs compared to those that reported missing no pills (35.58 vs. 31.31, *p* = 0.05).

### 3.1. Medication Adherence

Nearly one in five (17.9%) participants reported being nonadherent (missing at least one dose of CML medication in the last week) (on the BMQ—Table 2). On single-item measure one, 18.4% reported being nonadherent (missing at least one dose in the last week); on single-item adherence measure two, 16.3% reported their ability to take their CML medicine was less than excellent in the last 4 weeks. When comparing the two single item adherence questions, there was no significant difference by race for 7-day adherence. However, these results differed marginally between blacks/others who reported more difficulty taking their medicine as prescribed compared with whites (28.6% vs. 8.3%, *p* = 0.053) (Table 3). There was no significant difference regarding medication adherence by literacy level for the single item questions. Due to the small sample size, differences across sites were not assessed. 

Participants who reported that their ability to take CML medication in the last four weeks was excellent were less likely to report they had missed a dose than those who did not report excellent ability (87.5% vs. 50.0%, *p* = 0.003).

### 3.2. Medication Beliefs and Barriers

Almost half (43.0%) of participants felt their CML medicine was ineffective and 24.4% reported taking CML pills was somewhat to very hard. The two most common reasons for missing a dose were simply missed it (24.4%) and the side effects it caused (18.6%). Compared to participants who reported missing no doses of CML medication in the last 7 days, participants who missed at least one dose were significantly more likely to report that taking their medication was somewhat to very hard (50.0% vs. 18.6%, *p* = 0.008). They were also more likely to indicate they had missed doses because of side effects (50.0% vs. 11.4%, *p* = 0.001) or because of a busy schedule (37.5% vs. 2.9% *p* = 0.004). 

## 4. Discussion

We assessed self-reported OAC agent nonadherence among participants with CML across five community cancer clinics and characterized patient barriers to CML OAC adherence. To our knowledge, this is the first study to assess patient-reported medication adherence in CML across multiple community-based cancer centers. Our findings indicated almost all participants perceived their ability to take their CML medication was good to excellent, yet nearly one in five participants reported missing at least one dose in the last week. Of concern, one in four reported difficulties remembering to take their CML medication, and the main reason they reported missing doses was that they simply forgot, or they did not think their medication was working for them.

Our study findings were consistent with previous studies of CML adherence, with nearly 18% missing at least one dose of their CML medication in the previous 7 days. This puts patients at risk for falling below the threshold of 85% adherence necessary for a complete cytogenetic response. The nonadherence rate in this pilot study was consistent with that found by Ibrahim et al., which used the medication event monitoring system (MEMS) to measure adherence to imatinib in 87 participants [18,39]. They found that 26% of participants taking imatinib were less than 85% adherent. Moreover, compared to participants with an adherence rate of over 85%, less adherent participants had a higher probability of losing their cytogenetic response at two years (27% vs. 2%) and a lower probability of remaining on imatinib (65% vs. 92%). The authors concluded that poor adherence is the principal factor contributing to the loss of cytogenetic response and treatment failure in patients on long-term imatinib therapy. Efficace et al. [42] found only about half (53%) of CML participants taking TKIs reported strict adherence. Participants reported both intentional and unintentional reasons for nonadherence; the most common reason for each was dealing with side effects and forgetfulness. These were the two most common reasons given in our study with “simply missed it” or “missed it because of busy schedule” being almost twice as common as experiencing side effects from the medication.

There are varied reasons why patients may have suboptimal adherence to imatinib therapy. Some studies cited poorer patient understanding and knowledge of CML and its treatment, forgetfulness, concomitant drug burden, lower level of social support, depression, and financial burden as common reasons for medication nonadherence in CML patients [18,19,21,26,43,44]. In a recent qualitative study in Spain, Talens found barriers to OCA adherence included the impact of side effects on patients’ work, leisure time, and quality of life [45]. A Canadian survey found patients and providers have different perceptions of barriers to OAC agent adherence [44]. Most providers, but few patients, reported comprehension (92% vs. 1%), cost (91% vs. 25%), regimen complexity (88% vs. 4%), and interactions with other medications (76% vs. 21%) as barriers. Interestingly, almost all providers believed that patients reported adverse effects some or most of the time but 30% of patients indicated they never or rarely reported adverse effects [29,42,46]. 

Previous studies identified low patient health literacy as a barrier to patients’ clear understanding and adequate use of medication [47,48,49,50,51]. We anticipated this would be the case in our study, but this was not the case likely due to the small sample size. However, our finding that one in five participants had limited health literacy is not surprising. According to the only U.S. national health literacy survey to date, 14% of adults have below basic health literacy skills and another 22% have only basic skills [52]. Numerous studies have found limited health literacy is more prevalent among low-income patients cared for in community clinics [49,50,52,53,54,55,56,57,58,59,60]. This indicates the possibility that patient education and medication counseling could be enhanced by employing health literacy principles [61,62]. Those principles include use of plain language, easy-to-understand written materials focused on the benefits of taking their prescribed OAC medication and the risk of suboptimal adherence and asking patients to “teach-back” key information to confirm understanding. 

After testing the two single questions, we found only minor differences between the single-item questionnaires (assessing 1-week or 4-week adherence). Based on our previous medication adherence studies with HIV patients who had trouble remembering missed doses over 4 weeks [63], we believe it may be easier for patients to be asked to recall medication adherence over the past 7 days than recall the last 4 weeks and therefore more reliable. However, patient reports of behavior may be attributable to other factors such as taking medications prior to appointments to appear adherent.

This multisite pilot study demonstrates that the use of brief questionnaires is an easy no-cost method of assessing CML OAC adherence, beliefs, and barriers in busy community cancer clinics. Self-report, though not as specific as using pill counts, MEMS caps, or pharmacy fill data, is practical and efficient for use in busy clinics. A measurement of adherence that is complicated, expensive, intrusive, or time-consuming is not ideal in clinical settings [33]. Community cancer clinics having a simple means of regularly identifying suboptimal adherence could help identify at-risk patients for counseling. 

The single item adherence question “over the past seven days how many times did you miss a dose of your medication” has been used in previous chronic disease studies. Wu and colleagues found in a multisite randomized controlled trial that this single item self-report of medication adherence question predicted hospitalization and death over a year in patients with heart failure [33].

Thus, clinic staff could routinely ask patients the single item missed dose question to rapidly screen for adherence. If patient has missed a dose in the last week, staff could personalize the two belief questions from the BMQ ( how well does the CML medication work for you and how difficult is it to remember to take you CML medicine) and then to further tailor counseling give the 11-item medication adherence reasons scale to identify reasons for nonadherence. 

This study had some limitations, including the sample size and inclusion of English-speaking participants only. However, the study sites were geographically distributed, despite the study population being mostly white. To better understand medication adherence and barriers to medication-taking, more research is needed in community clinics that care for a greater number of low-income patients and patients from racial and ethnic minority groups who are more likely to have low health literacy [62]. Adherence was assessed by self-report and not by more stringent, but costly, methods such as the Medication Event Monitoring System (MEMS) caps [39], an objective measure of adherence that may not be suited for busy community oncology clinic settings. In addition, this pilot study was not designed to assess the validity and reliability of the assessment questions included in this study. That is an important area for future research. We also did not assess whether the type of health care provider (physician, nurse, pharmacist, medical assistant, etc.) administering the assessment questions would affect patient reporting and adherence findings All participants were taking at least one other prescription medication, but adherence to these medications was not assessed. 

## 5. Conclusions

Adherence to OAC agents prescribed for the treatment of CML is essential to maximize treatment effectiveness and clinical outcomes. We found adherence was suboptimal using a simple self-report question. The most common barriers to taking CML medication were “simply forgot” and side effects. If a patient reports missing a dose, clinic staff should administer the MARS to identify risk factors for nonadherence. Although more stringent methods of medication adherence may be ideal, they may not be feasible for busy community oncology clinics. The importance of these findings provides clinics with actionable insight to quickly identify patients at risk for nonadherence and screen for medication beliefs and barriers to personalize education and counseling. Future studies should assess adherence to all prescription medications taken by CML patients and expand the study population to include a greater number of participants, particularly minority patients. Future studies should assess adherence to all Rx meds taken by CML patients, expand the study populations to include a larger number of participants, particularly minority patients, and determine adherence relative to expected use among nonadherent patients to determine whether the level of nonadherence could potentially affect clinical outcomes (e.g., complete cytogenetic response). 

## Figures and Tables

**Table 1:** General demographics and medication adherence among patients with chronic myelogenous leukemia (CML) who were prescribed oral chemotherapy.

	Over the Past 7 Days, How Many Times Did You Miss a Dose of Your CML Medication?			
Demographics	Overall (n = 86)	0 Times (n = 70)	≥1 Time (n = 16)	*p*-Value
Age at registration (years)	58.67 ± 14.95 (57.24)	58.33 ± 14.91 (58.02)	60.16 ± 15.50 (55.33)	0.66
BMI (kg/m^2^)	32.05 (7.13) 30.96	31.31 (6.18) 30.74	35.28 (9.93) 33.76	0.05 *
	n (%)	n (%)	n (%)	*p*-value
Gender				0.10
Female	38 (44.2)	28 (40.0)	10 (62.5)	
Male	48 (55.8)	42 (60.0)	6 (37.5)	
Race				1.00
Non-Hispanic White	76 (88.4)	62 (88.6)	14 (87.5)	
Non-Hispanic Black	6 (7)	4 (5.7)	2 (12.5)	
Other	4 (4.7)	4 (5.7)	0 (0)	
Insurance				
VA care (yes)	1 (1.2)	1 (1.4)	0 (0)	N/A
Medicare (yes)	29 (33.7)	23 (32.9)	6 (37.5)	0.77
Medicaid (yes)	6 (7)	5 (7.1)	1 (6.3)	1.00
Private Insurance (yes)	65 (75.6)	53 (75.7)	12 (75.0)	1.00
Tricare (yes)	2 (2.3)	2 (2.9)	0 (0)	N/A
No Insurance (yes)	1 (1.2)	1 (1.4)	0 (0)	N/A
Other prescriptions				0.18
1	22 (25.6)	16 (22.9)	6 (37.5)	
2–5	39 (45.3)	35 (50.0)	4 (25.0)	
6+	25 (29.1)	19 (27.1)	6 (37.5)	
Any Comorbidities (yes)	60 (69.8)	49 (70.0)	11 (68.8)	1.00

* Significant *p* < 0.05.

**Table 2:** Medication Adherence Single Item Questions: past 7 days among patients with chronic myelogenous leukemia (CML) who were prescribed oral chemotherapy.

	Over the Past 7 Days, How Many Times Did You Miss a Dose of Your CML Medication?			
	Overall n = 86 n (%)	0 Times n = 70 n (%)	≥1 Times n = 16 n (%)	*p*-Value
Single Item adherence measures				
Last 4 weeks, ability to take CML medication				0.003 *
Excellent	72 (83.7)	63 (90.0)	9 (56.3)	
Non-Excellent	14 (16.3)	7 (10.0)	7 (43.8)	
Brief Medication Questionnaire (BMQ)				
Nonadherence (missed taking any pills)	15 (17.9)	1 (1.5)	14 (93.3)	<0.001 *
Medication Belief (how well does medication work for you—not well, NA or Do medications bother you—yes)	37 (43.0)	29 (41.4)	8 (50.0)	0.53
Recall (remember to take pill (Very hard, somewhat hard) or multi-dose—yes)	21 (24.4)	13 (18.6)	8 (50.0)	0.008 *
Rapid Estimate of Adult Literacy in Medicine short form (REALM-SF)				0.35
Adequate (7)	67 (77.9)	56 (80.0)	11 (68.8)	
Moderate (4–6)	16 (18.6)	11 (15.7)	5 (31.3)	
Low (0–3)	3 (3.5)	3 (4.3)	0 (0)	
Medications Adherence Reasons Scale (MARS) Form				
Experienced Side effects from this medication	16 (18.6)	8 (11.4)	8 (50.0)	0.001 *
Did not have the money to pay for the medicine	7 (8.1)	6 (8.6)	1 (6.3)	1.00
Have concerns about possible side effects from this medicine	4 (4.7)	3 (4.3)	1 (6.3)	0.57
Have concerns about long term effects from this medicine	8 (9.3)	5 (7.1)	3 (18.8)	0.16
Did not have the medicine because the pharmacy was out	4 (4.7)	4 (5.7)	0 (0)	1.00
Have trouble managing all the medicines I take	1 (1.2)	1 (1.4)	0 (0)	1.00
Would have taken the medicine but simply missed it	21 (24.4)	14 (20.0)	7 (43.8)	0.06
Would have taken it but missed it because of a busy schedule	8 (9.3)	2 (2.9)	6 (37.5)	0.004 *
Would have taken it but have problems forgetting things in my daily life	3 (3.5)	2 (2.9)	1 (6.3)	0.47

* Significant *p* < 0.05.

**Table 3:** Medication Adherence Single Item Questions: past 4 weeks among patients with chronic myelogenous leukemia (CML) who were prescribed oral chemotherapy.

	Last 4 Weeks, Ability to Take CML Medication			
Single Item Adherence Measures	Overall n = 86 n (%)	Excellent n = 72 n (%)	Non-Excellent n = 14 n (%)	*p*-Value
Race				0.05
Non-Hispanic White	76 (88.4)	66 (91.7)	10 (71.4)	
Non-Hispanic Black	6 (7.0)	3 (4.2)	3 (21.4)	
Other	4 (4.7)	3 (4.2)	1 (7.1)	
Over the past 7 days, how many times did you miss a dose of your CML medication				0.003 *
0	70 (81.4)	63 (87.5)	7 (50.0)	
1	11 (12.8)	7 (9.7)	4 (28.6)	
2	1 (1.1)	0 (0)	1 (7.1)	
3	1 (1.1)	0 (0)	1 (7.1)	
7	3 (3.5)	2 (2.8)	1 (7.1)	
Over the past 7 days, how many times did you miss a dose of your CML medication				0.003 *
0	70 (81.4)	63 (87.5)	7 (50.0)	
≥1	16 (18.6)	9 (12.5)	7 (50.0)	
Brief Medication Questionnaire (BMQ)				
Regimen Screen (miss taking any pills—yes)	15 (17.4)	7 (10.0)	8 (57.1)	<0.001 *
Belief Screen (how well does medication work for you—not well, NA or Do medications bother you—yes)	37 (43.0)	28 (38.9)	9 (64.3)	0.08
Recall Screen (remember to take pill (Very hard, somewhat hard) or multi-dose—yes)	21 (24.4)	16 (22.2)	5 (35.7)	0.28
Rapid Estimate of Adult Literacy in Medicine short form (REALM-SF)				1.00
Adequate (7)	67 (77.9)	56 (77.8)	11 (78.6)	
Moderate (4–6)	16 (18.16	13 (18.1)	3 (21.4)	
Low (0–3)	3 (3.5)	3 (4.2)	0 (0)	
Medications Adherence Reasons Scale (MARS) Form				
Experienced Side effects from this medication	16 (18.6)	13 (18.1)	3 (21.4)	0.72
Did not have the money to pay for the medicine	7 (8.1)	7 (9.7)	0 (0)	0.59
Do not think I need this medication anymore	0 (0)	0 (0)	0 (0)	N/A
Do not think this medication is working for me	0 (0)	0 (0)	0 (0)	N/A
Have concerns about possible side effects from this medicine	4 (4.7)	3 (4.2)	1 (7.1)	0.52
Have concerns about long term effects from this medicine	8 (9.3)	7 (9.7)	1 (7.1)	1.00
Did not have the medicine because the pharmacy was out	4 (4.7)	2 (2.8)	2 (14.3)	0.12
Have trouble managing all the medicines I take	1 (1.2)	1 (1.4)	0 (0)	1.00
Would have taken the medicine but simply missed it	21 (24.4)	10 (13.9)	11 (78.6)	<0.001 *
Would have taken it but missed it because of a busy schedule	8 (9.3)	3 (4.2)	5 (35.7)	0.002 *
Would have taken it but have problems forgetting things in my daily life	3 (3.5)	2 (2.8)	1 (7.1)	0.42

* Significant *p* < 0.05.

## Data Availability

The datasets generated during and/or analyzed during the current study are not publicly available due to the data set containing PHI but are available from the corresponding author on reasonable request.
